# Metabolic responses to albumin deficiency differ distinctly between partial and full ablation of albumin expression in mice

**DOI:** 10.1186/s12944-024-02229-3

**Published:** 2024-08-09

**Authors:** Afsoun Abdollahi, Mirandia Szramowski, Keigo Tomoo, Gregory C. Henderson

**Affiliations:** Department of Nutrition Science, Purdue University, STON 208, 700 Mitch Daniels Blvd, West Lafayette, IN 47907 USA

**Keywords:** Non-esterified fatty acid, Hypoalbuminemia, Lipid, Energy metabolism

## Abstract

It had been observed that homozygous albumin knockout mice (*Alb*^−/−^) exhibit low plasma free fatty acid (FFA) concentration and improved blood glucose regulation. However, it was not yet known to what extent heterozygous albumin knockout (*Alb*^+/−^) mice would display a similar phenotype. *Alb*^−/−^, *Alb*^+/−^, and wild-type (WT) female mice were studied on a low-fat diet (LFD) or high-fat diet (HFD). On both diets, decreased plasma FFA concentration, and improved glucose tolerance test were observed in Alb^−/−^, but not in *Alb*^+/−^, compared to WT. Plasma adiponectin concentration showed greater elevation in *Alb*^−/−^ than *Alb*^+/−^. Consistent with that, adiponectin gene expression was significantly higher in *Alb*^−/−^ mice than in *Alb*^+/−^ and WT mice. A dose-dependent response was observed for hepatic *Acadl* gene expression showing higher *Acadl* gene expression in *Alb*^−/−^ mice than in *Alb*^+/−^ and WT mice. In conclusion, although female *Alb*^+/−^ mice exhibited some slight differences from WT mice (e.g., increased plasma adiponectin and hepatic *Acadl* gene expression), *Alb*^+/−^ mice did not exhibit improved glucoregulation in comparison to WT mice, indicating that a minor suppression of albumin expression is not sufficient to improve glucoregulation. Furthermore, it is now clear that although the response of female mice to HFD might be unique from how males generally respond, still the complete albumin deficiency in *Alb*^−/−^ mice and the associated FFA reduction is capable of improving glucoregulation in females on this diet. The present results have implications for the role of albumin and FFA in the regulation of metabolism.

## Introduction

Obesity increases the risk of developing insulin resistance, and insulin resistance is associated with elevated lipolysis and increased free fatty acid (FFA) flux in the bloodstream [1]. It has been shown that increased plasma FFA concentration is associated with an increased risk of developing multiple chronic diseases such as cardiovascular disease [2], non-alcoholic fatty liver disease [3], and type 2 diabetes [4, 5]. Fluctuations in plasma FFA concentration are known to alter glucoregulation [6], and therefore the connection between plasma FFA and glucose metabolism is of great interest.

In hopes of decreasing the burden of these diseases through plasma FFA reduction, researchers have studied multiple pathways such as sequestering FFA as triacylglycerol (TAG) in adipose tissue through lipolysis inhibition or membrane FFA transporter disruption. Whole-body adipose triglyceride lipase (ATGL) knockout mice had decreased plasma FFA concentration and improved glucoregulation; however, these mice had increased ectopic lipid deposition in tissues such as the liver, muscle, and heart. The severe cardiomyopathy in these mice led to premature death [7]. Sequestering FFA only in adipose tissue might protect the mice against ectopic lipid deposition. Adipose-specific ATGL knockout mice exhibited decreased plasma FFA concentration, lower hepatic lipid accumulation, and improved insulin signaling and glucose metabolism [8]. A membrane FFA transport reduction study has shown that fatty acid transporter-1 (FATP1) null mice were resistant to diet-induced obesity and insulin resistance [9]. Another study demonstrated that mice with a loss of fatty acid transporter-2 (FATP2) in the liver had improved insulin sensitivity and glucose metabolism [10]. In addition to sequestering FFAs in adipose tissue, and decreasing FFA uptake by hepatocytes, targeting FFA transport in the bloodstream can be another approach for metabolic health improvement. We previously showed that albumin deficient (*Alb*^−/−^) mice exhibited lower levels of plasma FFA, hepatic lipids, and blood glucose than observed in wild-type (WT) mice when the animals were fed a chow diet [11]. FFA transport interruption through albumin deficiency also illustrated that male mice on both a low-fat diet (LFD) and a high-fat diet (HFD) had decreased hepatic lipid accumulation and improved glucoregulation through potential mechanisms including increased expression of peroxisome proliferator-activated receptor-α in liver and adipose tissue, as well as elevated expression of adiponectin and glucose transporter-4 in adipose tissue [12]. Additionally, we showed that the lower level of plasma FFA in *Alb*^−/−^ mice compared to the WT mice is due to a decreased appearance rate of FFA in the bloodstream [13].

The magnitude of albumin deficiency that is required for metabolic health enhancement remained undiscovered. It was expected that heterozygous knockout (*Alb*^+/−^) would lead to approximately 30% suppression of albumin expression [14]. Each albumin molecule has seven binding sites for FFA [15], and the serum albumin expression is around 0.2–0.4 mM in mice [16, 17]. Thus, the total FFA binding capacity of serum albumin in WT mice might be around 1.4–2.8 mM FFA, well beyond the typical physiological range. Therefore, it was conceivable that ~ 30% reduction in plasma albumin concentration would not reduce plasma FFA concentration and thus might have minimal effects on fatty acid metabolism. We were interested in studying *Alb*^+/−^ mice, since it was not investigated previously if ~ 30% albumin reduction would suffice to see the metabolic health improvements. It was also not known if female mice would show similar metabolic health improvements as seen in males on a HFD previously [12], as female mice may be less susceptible to HFD-induced metabolic dysfunction [18–21]. Therefore, the effects of albumin deficiency in obese animals might differ from the results seen previously in males, and it remained critical to understand how females on a HFD respond to albumin deficiency. We studied the metabolic response of *Alb*^+/−^ and *Alb*^−/−^ female mice to a HFD as compared to a LFD. By using *Alb*^+/−^ and *Alb*^−/−^ mice to study partial and full ablation of albumin expression, we investigated the effect of different albumin levels on determining plasma FFA concentrations and their impact on improving glucose metabolism.

## Materials and methods

### Mouse model

The institutional animal care and use committee of Purdue University approved this protocol. Female mice on the C57BL/6J genetic background were investigated in this study, with intact albumin expression or one of two levels of whole-body albumin gene knockout (heterozygous versus homozygous knockout). The development of this albumin knockout model has been described in the literature (29), and the mice for the present study were purchased from the Jackson Laboratory (Bar Harbor, ME). WT (strain 000664), as well as *Alb*^+/−^ (strain 025200) and *Alb*^−/−^ mice (strain 025200) were studied.

### Animal study overview

Female WT, *Alb*^+/−^, and *Alb*^−/−^ mice, each fed a LFD or a HFD (n = 5–7 per group), were housed in the animal facility with a 12-h light/dark photoperiod. Mice were allowed ad libitum access to food and water except for a brief withdrawal of food before specific procedures as described below. The mice were fed the LabDiet 5K52 diet (Land O’ Lakes, Inc, Richmond, IN, USA) until 10 weeks of age, and then they were assigned to receive the LFD (D12450J) or HFD (D12492) (Research Diets, New Brunswick, NJ) for eight weeks. LFD contained 10% of energy from fat, and HFD contained 60% of energy from fat. At 18 weeks of age, the phenotyping assessments (described below) had reached completion, and then tissues were collected following euthanasia. Food was weighed weekly for monitoring food intake.

### Bomb calorimetry

As an indicator of nutrient absorption, a bomb calorimeter was used to determine the energy content of mouse feces (cal/g). This was determined because elevations in energy content might indicate nutrient malabsorption. Fecal samples were collected over a period of 7 days and placed in aluminum foil trays. Subsequently, the samples were dried in a drying oven at 105 °C for 24 h. Each sample, weighing approximately 600 mg, was then loaded into the bomb calorimeter (Parr Instrument Company, Moline, IL, USA), with each run performed in duplicate to determine the energy content of the mouse feces.

### Indirect calorimetry

At 16 weeks of age, oxygen consumption (VO_2_) and carbon dioxide production (VCO_2_) were measured in cages equipped for analysis of expired air from mice (Columbus Instruments, Columbus, OH, USA) [22]. First the mice were acclimatized to the metabolic cages for approximately 24 h. Next, data were collected over a 24-hour period. In total, mice were in the metabolic cages for 2 days with data collected on the second day. Standard equations were used to calculate energy expenditure using VO_2_ and the calculated respiratory quotient (RQ). Food and water were available to the mice while they were in the metabolic cages.

### Oral glucose tolerance test

At 17 weeks of age, OGTT was performed by using orogastric gavage to administer a glucose solution (2 g/kg body weight). The glucose solution was prepared at a 20% (wt/vol) concentration in water. At each time point, a small drop of blood from the tail was discarded followed by drawing blood from that same site for glucose analysis. In duplicate, glucose was measured with a point-of-care device (Prodigy Diabetes Care, Charlotte, NC, USA) at times 0, 10, 20, 30, 60, 90, and 120 min. The area under the curve (AUC) was calculated as a Riemann Sum (i.e., the trapezoidal rule). The mice were fasted 4–6 h before the test.

### Tissue collection

The animals were fasted for 4–6 h before the euthanasia by CO_2_ inhalation. Blood was then collected by cardiac puncture and transferred to ethylenediaminetetraacetic acid (EDTA) tubes, kept on wet ice, and centrifuged at 3000 x g for 15 min to obtain plasma. The liver and perigonadal adipose tissue were quickly collected and then submerged in liquid nitrogen, then stored frozen at − 80 °C until analysis.

### Biochemical assays

Heptane, isopropanol, and 0.033 N sulfuric acid were used for the extraction of lipids from plasma with addition of heptadecanoic acid (17:0) as the internal standard. Following vigorous shaking, the extraction mixes were centrifuged at 700 x g to separate the phases. Next, the heptane extracts were removed, dried, and then reconstituted in sample diluent for analysis of FFA by liquid chromatography/mass spectrometry (LC-MS) using a model 1260 LC and model 6160 single‐quadrupole MS (Agilent Technologies, Santa Clara, CA, USA); reversed phase separation and selective ion monitoring were employed to quantitate the internal standard, myristic acid (14:0), myristoleic acid (14:1), palmitic acid (16:0), palmitoleic acid (16:1), stearic acid (18:0), oleic acid (18:1), linoleic acid (18:2), α‐linolenic acid (18:3), arachidonic acid (20:4), eicosapentaenoic acid (20:5), and docosahexaenoic acid (22:6) following our established method [23–25]. Total cholesterol (TC) and high-density lipoprotein cholesterol (HDL-C) were measured by automated assays, and insulin was measured by enzyme-linked immunosorbent assay (Mercodia, Minneapolis, MN, USA), each of these assays carried out at Indiana University School of Medicine in the Translation Core Facility. For TAG concentration measurement, homogenized liver tissue was extracted with heptane, isopropanol, and water, followed by a colorimetric assay that was carried out on the lipids that were in the heptane phase of the extraction, as described previously [11]. Plasma adiponectin was measured using a mouse adiponectin/Acrp30 Quantikine ELISA kit (R&D systems, Minneapolis, MN, USA).

### Plasma albumin analyses

Albumin deficiency was previously demonstrated in *Alb*^−/−^ mice through mass spectrometry analysis [11]. To confirm this expectation in the present study, sodium dodecyl sulfate–polyacrylamide gel electrophoresis **(**SDS-PAGE) was used. Plasma samples were diluted 40-fold or 500-fold with gel loading buffer containing Laemmli Sample Buffer (Biorad, Hercules, CA, USA) and β-mercaptoethanol. 20 µl of 40-fold diluted plasma was run by SDS-PAGE for qualitative visualization of the range of protein molecular weights in plasma. For the purpose of quantitatively estimating albumin expression, SDS-PAGE was carried out with lower protein loading to minimize saturation of the intensity in the albumin band; in this case, 10 µl of 500-fold diluted plasma was loaded. Plasma proteins were separated by 4–15% Tris–HCl precast gel (BioRad), with a human albumin standard as a reference. The gel electrophoresis was done with a voltage set at 100 V for 10 min and at 200 V for approximately 45 min until the loading dye migrated near the gel’s base. After that, the gel was stained by Coomassie Brilliant Blue R-250 Staining Solution (Biorad) for one hour, followed by destaining with Coomassie Brilliant Blue R-250 Destaining Solution (Biorad). The resulting gels were then scanned using an Odyssey CLx Imager (LI-COR Biosciences, Lincoln, NE, USA) and the band intensity at approximately 60–70 kD was quantitated for approximation of albumin expression.

### mRNA quantitation

A bead homogenizer was used to homogenize adipose tissue (~ 100 mg per mouse) and liver (~ 10 mg per mouse). Following this homogenization in Trizol Reagent, kits were used to isolate RNA (Qiagen, Hilden, Germany), 100 ng/µL RNA was used for cDNA synthesis (Agilent Technologies), and then qPCR analysis was carried out in triplicate for each sample using a Real-Time PCR System (Applied Biosystems) with Taqman primers and probes (Table 1). 3.4 ng cDNA was loaded per well. The mRNA for the genes of interest was normalized to 18 S ribosomal RNA. The comparative Ct method was used to calculate the final gene expression results, and the data were further normalized such that the average for WT mice on LFD would be equal to a value of 1.

**Table 1:** Primers and probes for qPCR with Thermo Fisher catalog numbers for the assays. Abbreviations: *acs* (acyl CoA synthase), *Glut2* (glucose transporter-2), *Tlr4* (toll-like receptor-4), *Acadl* (Acyl-CoA dehydrogenase long chain), *Acox1* (Acyl-CoA oxidase 1)

Gene	Assay ID	Probe Catalog number
*18 S*	Hs99999901_s1	4448484
*Acs*	Mm00484217_m1	4331182
*Glut2*	Mm00446229_m1	4331182
Adiponectin	Mm00456425_m1	4331182
*Tlr4*	Mm00445273_m1	4331182
*Acadl*	Mm00599660_m1	4331182
*Acox1*	Mm01246834_m1	4331182

### Western blot

Liver samples were prepared as described previously [11]. Briefly, 30–40 mg of the liver was homogenized, then samples were centrifuged at 10,000 x g for 15 min at 4 °C. Samples were heated at 95 °C for 5 min to denature proteins. 50 µg of protein for each sample was loaded into each well. Samples underwent gel electrophoresis on a 4–15% polyacrylamide gel, then transferred to nitrocellulose membranes (BioRad). The membranes were blocked with 5% nonfat dried milk. Primary antibodies (1:1000 dilutions; Cell Signaling Technology, Danvers, MA, USA) were against NF-κB (p65) (catalog #8242S), phosphorylated NF-κB (phospho-p65) (catalog #3036S), AMPKα (catalog #5831), phosphorylated AMPKα (catalog #50081), and β-actin (catalog #4967S). IR Dye 680 or IR Dye 800 (LI-COR Biosciences), each at a 1:15,000 dilution, was used as the secondary antibody. The approach for incubating the membranes with antibody solutions, and the washing steps, have been described previously [11]. Membrane imaging was performed with an Odyssey CLx Imager (LI-COR Biosciences). The band intensities for proteins of interest were normalized to the intensity of β-actin bands, and the data were further normalized such that the average for WT mice on LFD would be equal to a value of 1.

### Statistical analysis

Data are presented as means ± SE. Analysis of variance (ANOVA) was used to analyze the results; genotype (with three levels including *Alb*^−/−^, *Alb*^+/−^, and WT) and diet (with two levels including LFD and HFD) were the factors in the analysis, as well as time being added as an additional factor for certain data as was appropriate. Additionally, analysis of covariance (ANCOVA) was used for the analysis of VO_2_ and metabolic rate, with covariate adjustment for body weight. Post hoc testing, when warranted, was achieved using Fisher’s least significant difference test. JMP Software (SAS Institute Inc., Cary, NC, USA) was used for running the statistical analyses, and the threshold for statistical significance was *p* ≤ 0.05.

## Results

**Fig. 1:**
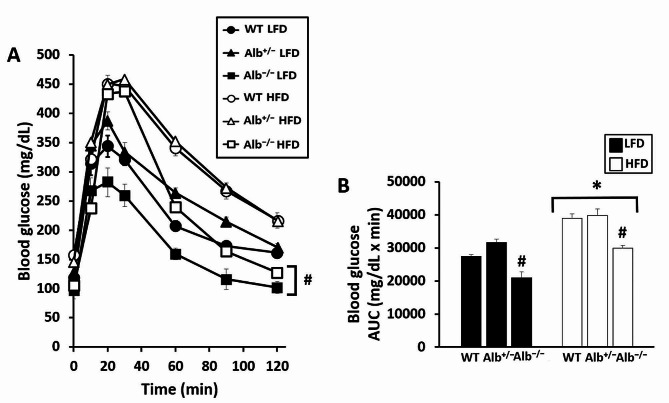
Oral glucose tolerance test (OGTT). Blood glucose during the OGTT (**A**). Blood glucose area under the curve (AUC) for blood glucose concentration (**B**). ^#^ Alb^−/−^ different from WT and Alb^+/−^ within the diet (*p* < 0.05). * HFD different from LFD (main effect of diet, *p* < 0.05). *n* = 5 − 7. Analysis by ANOVA. The values are expressed as mean and standard error

In order to investigate different levels of albumin on blood glucose regulation, OGTT was performed. In the testing of blood glucose regulation, for OGTT there was a main effect of genotype (*p* < 0.05), a main effect of diet (*p* < 0.05), and a main effect of time (*p* < 0.05) (Fig. 1A). There were no interactions between factors. Post hoc analysis of genotypes indicated that *Alb*^−/−^ displayed lower blood glucose levels than *Alb*^+/−^ and WT, and *Alb*^+/−^ was not significantly different from WT. Glucose AUC analysis showed that there was a main effect of genotype (*p* < 0.05) and a main effect of diet (*p* < 0.05) (Fig. 1B). There was no genotype-by-diet interaction. Post hoc analysis of genotype showed that blood glucose was lower in *Alb*^−/−^ than *Alb*^+/−^, with no significant difference between *Alb*^+/−^ and WT. There were no significant differences between the genotypes for food intake and body composition (Table 2). In order to investigate whether mice with various genotypes exhibited different levels of macronutrient absorption during digestion, we measured the fecal energy content. There was no main effect of genotype for fecal energy content; however, there was a main effect of diet (*p* < 0.05), indicating mice on HFD had higher energy content in their feces (Table 2). For plasma insulin, there was a main effect of genotype, a main effect of diet, and genotype-by-diet interaction. Post hoc analysis showed that plasma insulin levels were significantly lower in *Alb*^−/−^ than *Alb*^+/−^ and WT mice on HFD, and it was similar to the level of insulin in mice on LFD (*p* < 0.05) (Table 2).

**Table 2:** Animal characteristics. ^^^ Significantly different from WT within the diet, *p* < 0.05. ^#^ Significantly different from WT and Alb^+/−^ within the diet, *p* < 0.05. * LFD significantly different from HFD (main effect of diet), *p* < 0.05. TC, Total cholesterol concentration in plasma; HDL-C, High-density lipoprotein cholesterol concentration in plasma; TAG, Triacylglycerol concentration in plasma. *n* = 5–7 mice per group. Analysis by ANOVA. Values are means ± S.E

	LFD			HFD		
	WT	Alb^+/−^	Alb^−/−^	WT	Alb^+/−^	Alb^−/−^
Food Intake (g/d)	2.92 ± 0.09	3.06 ± 0.02	3.04 ± 0.07	3.03 ± 0.23	2.84 ± 0.13	2.87 ± 0.41
Body Weight* (g)	20.9 ± 0.4	20.2 ± 0.4	21.1 ± 0.5	26.5 ± 1.5	26.9 ± 1.4	23.5 ± 1.2
Body Fat* (%)	7.1 ± 0.6	6.5 ± 0.5	9.5 ± 1.1	22.3 ± 3.8	26.7 ± 3.0	19.1 ± 2.7
Feces energy (cal/g) *	3451.4 ± 12.4	3424.6 ± 17.3	3421.2 ± 21.7	3722.9 ± 33.1	3731.2 ± 23.6	3744.2 ± 32.0
Insulin (µg/L) *	0.60 ± 0.04	0.68 ± 0.05	0.43 ± 0.07	1.13 ± 0.13	1.00 ± 0.10	0.44 ± 0.06 ^#^
TC (mM)*	2.4 ± 0.1	3.0 ± 0.1^	3.8 ± 0.2^	3.2 ± 0.4	4.4 ± 0.1^	4.7 ± 0.2^
HDL-C (mM)*	0.97 ± 0.05	1.06 ± 0.03	0.83 ± 0.18	1.23 ± 0.16	1.61 ± 0.05	1.45 ± 0.09

**Fig. 2:**
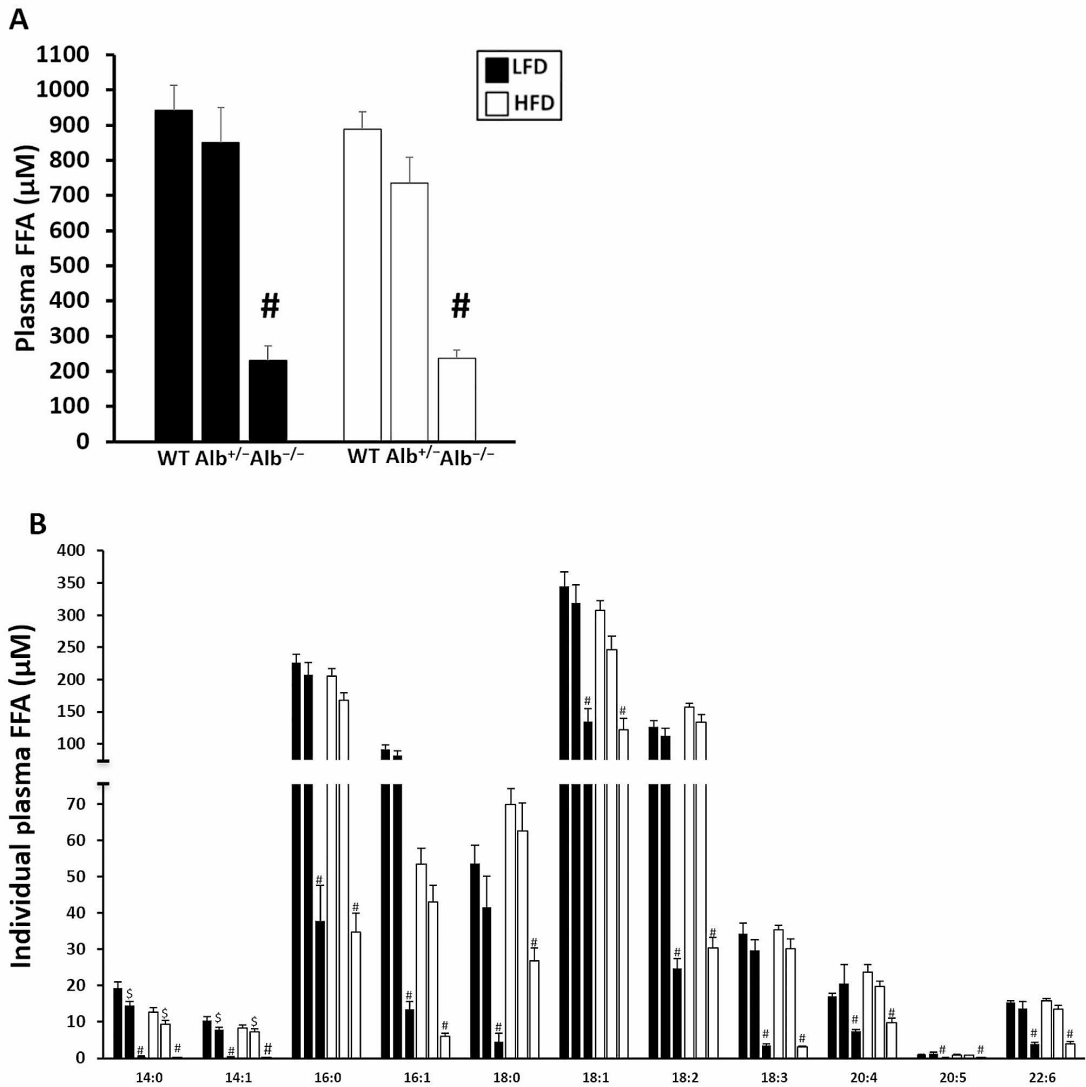
Total (**A**) and individual (**B**) plasma free fatty acid (FFA) concentration. The individual FFA species that were measured were myristic acid (14:0), myristoleic acid (14:1), palmitic acid (16:0), palmitoleic acid (16:1), stearic acid (18:0), oleic acid (18:1), linoleic acid (18:2), α-linolenic acid (18:3), arachidonic acid (20:4), eicosapentaenoic acid (20:5), and docosahexaenoic acid (22:6). ^#^ Alb^−/−^ different from Alb^+/−^ and WT within a diet (*p* < 0.05), ^$^ Alb^+/−^ different from WT and Alb^−/−^ within a diet (*p* < 0.05). *n* = 5 − 6. Analysis by ANOVA. The values are expressed as mean and standard error

**Fig. 3:**
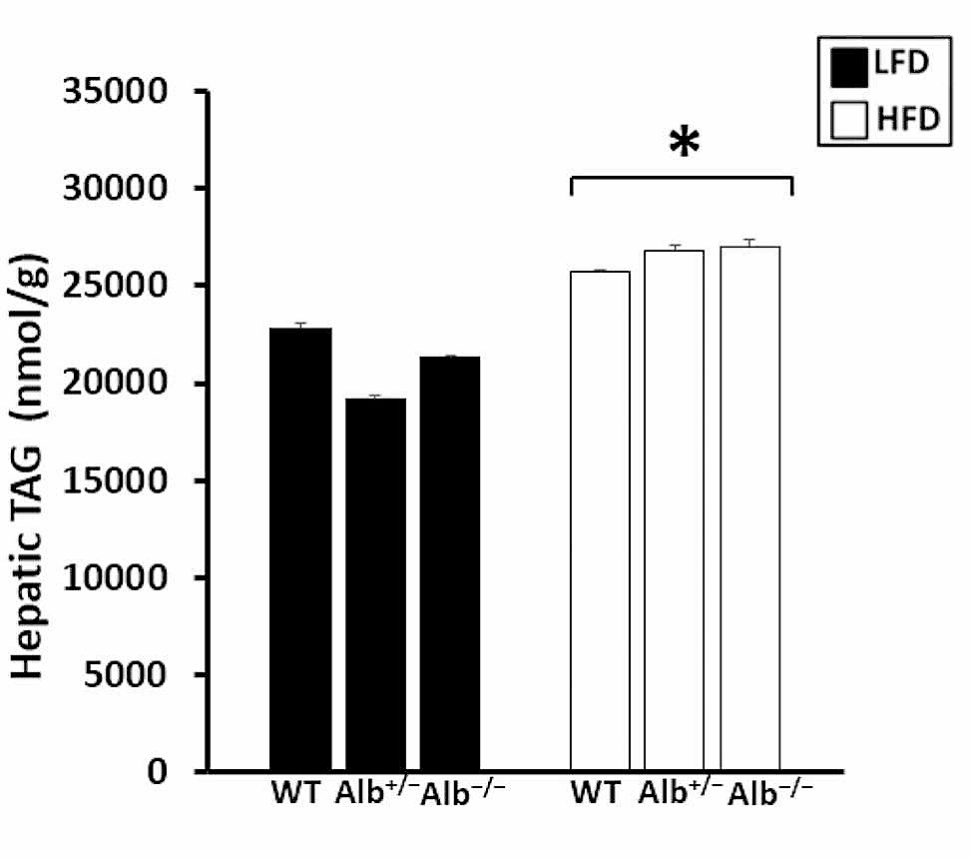
Triacylglycerol (TAG) concentration in the liver. * HFD different from LFD (main effect of diet, *p* < 0.05). *n* = 5 − 6. Analysis by ANOVA. The values are expressed as mean and standard error

As albumin plays a major role in FFA transport in bloodstream, we measured the level of plasma FFA in response to albumin reduction. Plasma FFA analysis showed that there was a main effect of genotype (*p* < 0.05). There was no main effect of diet nor genotype-by-diet interaction. The post hoc analysis of genotype revealed that FFA levels were significantly lower in *Alb*^−/−^ than *Alb*^+/−^ and WT with no significant difference between *Alb*^+/−^ and WT (Fig. 2A). Each measured individual FFA (14:0, 14:1, 16:0, 16:1, 18:0, 18:1, 18:2, 18:3, 20:4, 20:5, 22:6) showed a main effect of genotype (*p* < 0.05), and the pattern of results for individual FFA species (Fig. 2B) was generally similar to total FFA. There was a main effect of genotype (*p* < 0.05) and a main effect of diet (*p* < 0.05) for plasma TC, and there was no genotype-by-diet interaction. Post hoc analysis indicated that the TC concentration in plasma was significantly higher (*p* < 0.05) in *Alb*^−/−^ and *Alb*^+/−^ than WT on both diets. Mice on HFD had significantly higher levels of TC (*p* < 0.05). There was neither a main effect of genotype nor genotype-by-diet interaction for plasma HDL-C (Table 2). There was a main effect of diet (*p* < 0.05) and post hoc analysis showed that mice on HFD had increased HDL-C compared to the mice on LFD. There was no main effect of genotype on body weight or body fat percentage. There was a main effect of diet (*p* < 0.05) on both body weight and body fat percentage, with mice on HFD diet having significantly higher body weight and body fat percentage than mice on the LFD (Table 2). For hepatic TAG there was a main effect of diet but no main effect of genotype nor genotype-by-diet interaction (Fig. 3).

**Fig. 4:**
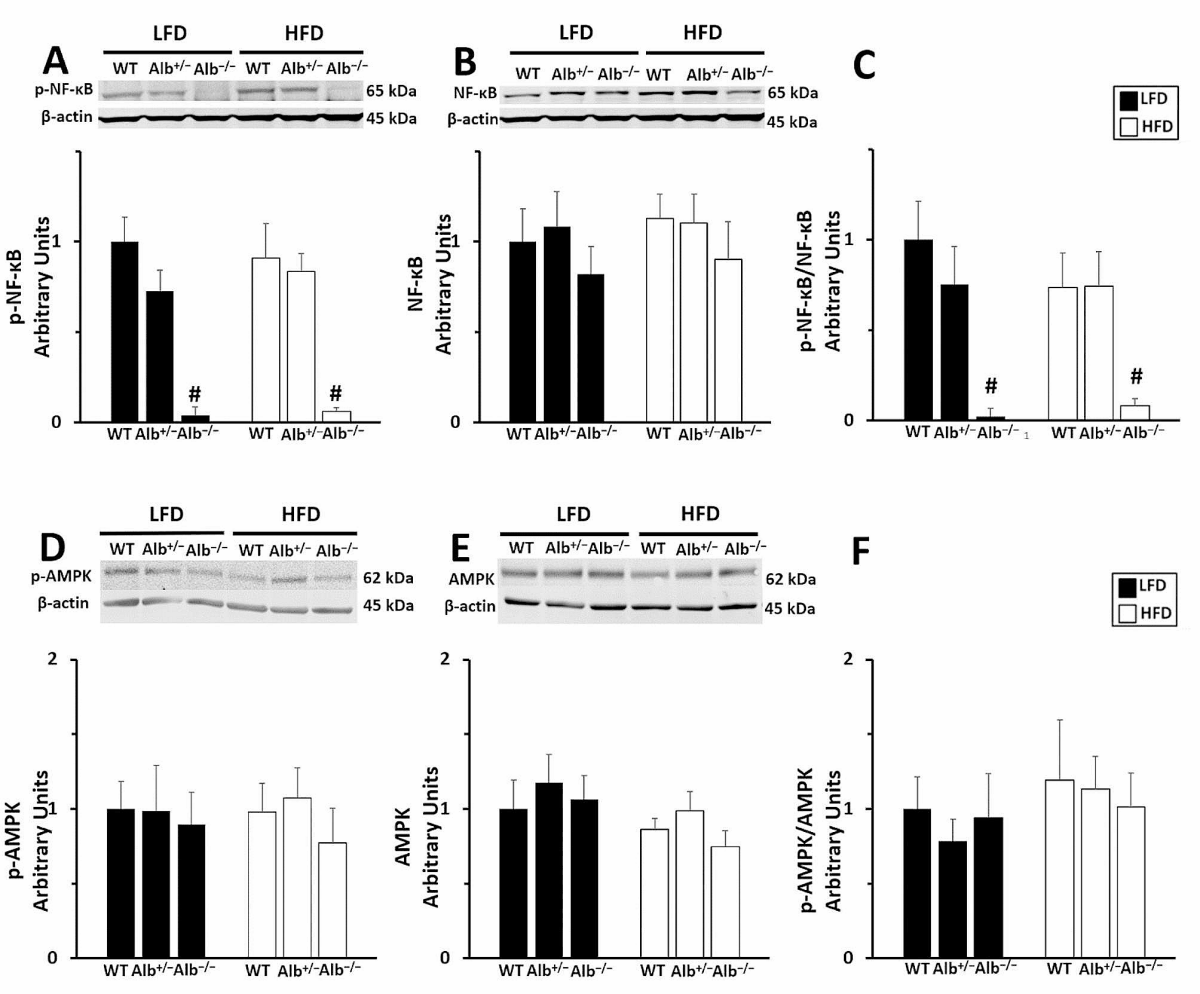
Western blot in the liver. Nuclear Factor kappa B (NF*-*κB) (**A**-**C**). Phosphorylated NF-κB (p65 subunit*)* (**A**), total NF-κB (**B**), the ratio of phosphorylated to total NF-κB (**C**). AMPKα in the liver (**D**-**F**). Western blot for phosphorylated AMPKα (**D**), total AMPKα (**E**), and the ratio of phosphorylated to total AMPKα (**F**). ^#^ Alb^−/−^ different from Alb^+/−^ and WT within a diet (*p* < 0.05). Band intensity normalized to β-actin. WT group on the LFD is set to a value of 1. *n* = 5 − 6 mice per group. Analysis by ANOVA. The values are expressed as mean and standard error

FFAs are a ligand for the TLR4/ NF-κB signaling pathway. As a response to the finding of FFA reductions in plasma, we measured total NF-κB, and the activated form of NF-κB. For phosphorylated NF-κB protein (p65 subunit) and its ratio to total NF-κB protein in liver, there was a main effect of genotype (*p* < 0.05), no main effect of diet, and no genotype-by-diet interaction. Post hoc analysis indicated that phosphorylated NF-κB and its ratio to total NF-κB were lower in *Alb*^−/−^ than in *Alb*^+/−^ and WT with no significant difference between *Alb*^+/−^ and WT (Fig. 4A, C). The total NF-κB in the liver was not significantly different between groups (Fig. 4B). Phosphorylated AMPKα and total AMPKα in the liver were not significantly different between genotypes (Fig. 4D-F).

**Fig. 5:**
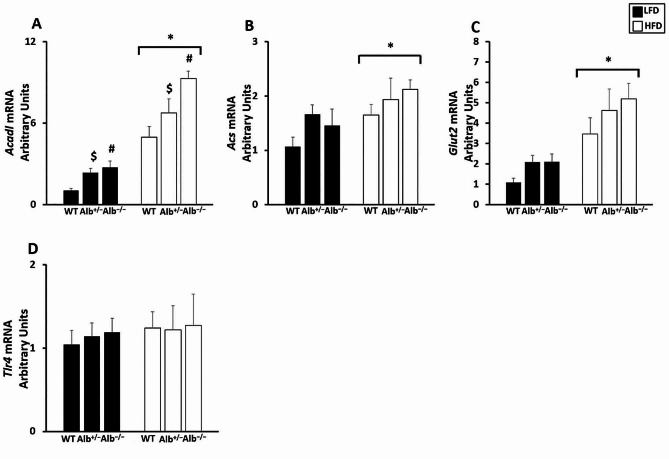
Gene expression in the liver. ^#^ Alb^−/−^ different from Alb^+/−^ and WT within a diet (*p* < 0.05), ^$^ Alb^+/−^ different from WT and Alb^−/−^ within a diet (*p* < 0.05), * HFD different from LFD (*p* < 0.05). *n* = 5 − 6. mRNA for the genes of interest was normalized to 18 S. WT group on the LFD is set to a value of 1. Analysis by ANOVA. The values are expressed as mean and standard error

**Fig. 6:**
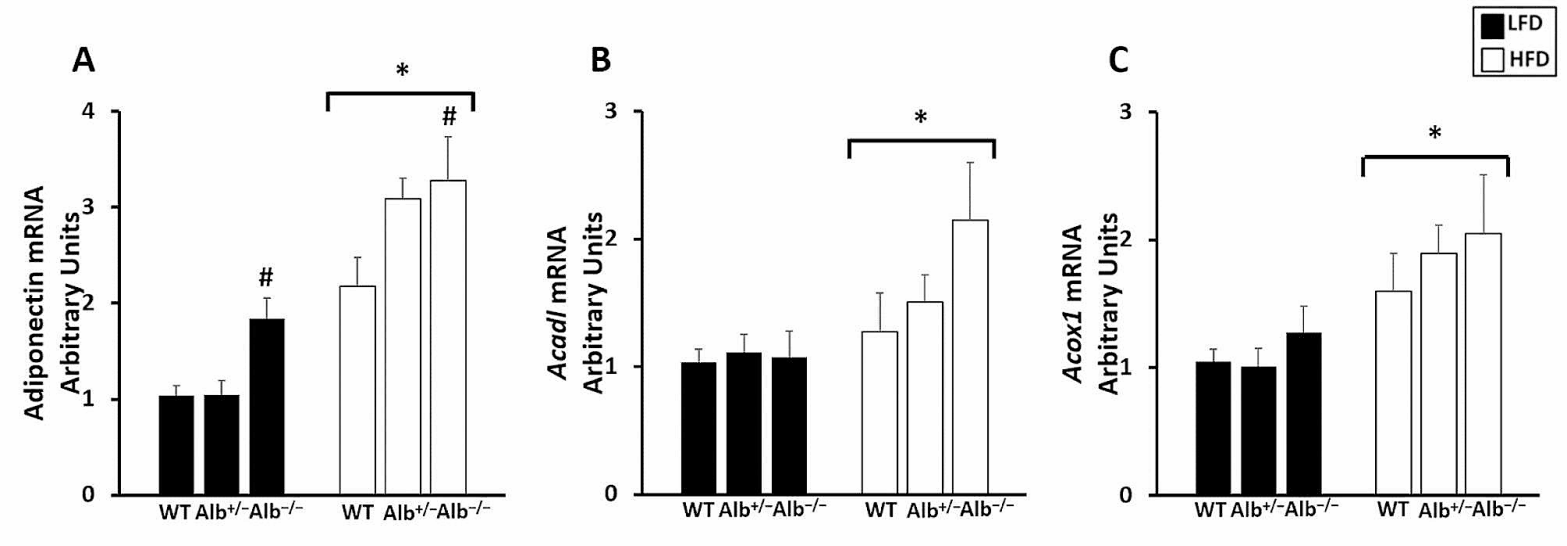
Gene expression in adipose tissue. ^#^ Alb^−/−^ different from Alb^+/−^ and WT within a diet (*p* < 0.05), * HFD different from LFD (*p* < 0.05). *n* = 5 − 6. mRNA for the genes of interest was normalized to 18 S. WT group on the LFD is set to a value of 1. Analysis by ANOVA. The values are expressed as mean and standard error

**Fig. 7:**
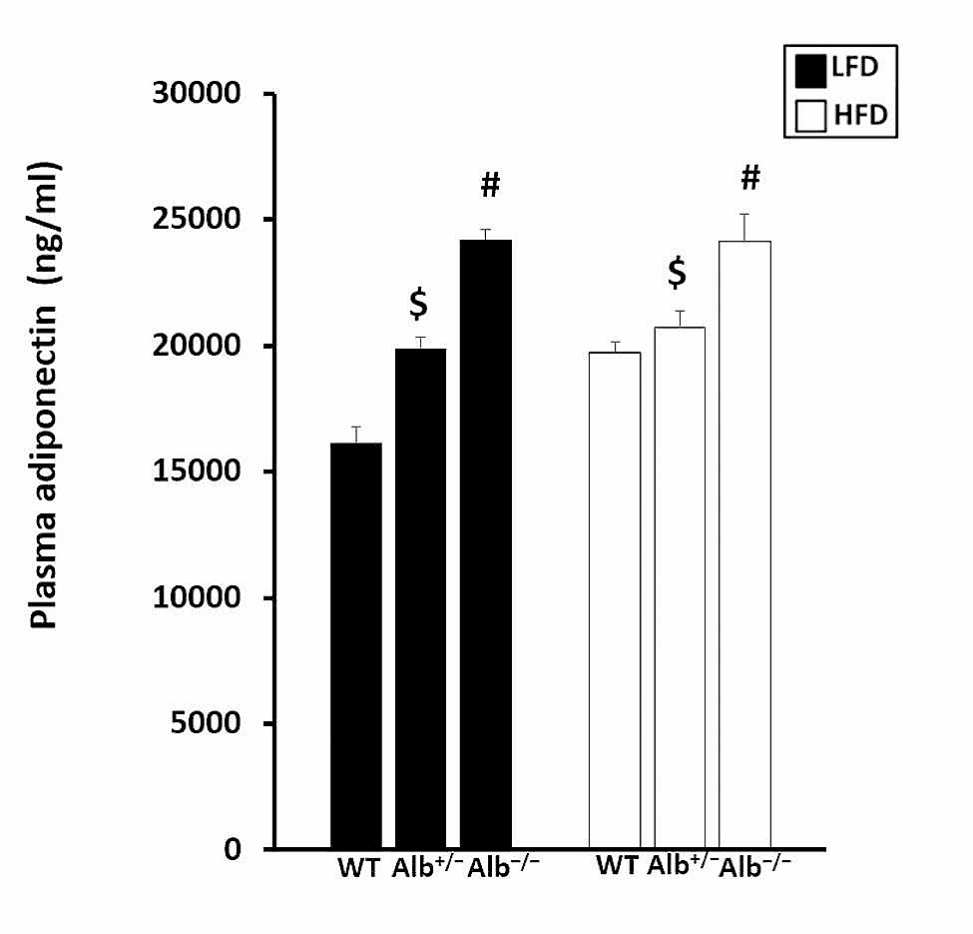
Plasma adiponectin concentration (ng/ml). ^#^ Alb^−/−^ different from Alb^+/−^ and WT within a diet (*p* < 0.05), ^$^ Alb^+/−^ different from WT and Alb^−/−^ within a diet (*p* < 0.05). *n* = 5 − 6. Analysis by ANOVA. The values are expressed as mean and standard error

In order to investigate liver and adipose tissue responses to the plasma albumin concentration changes, we studied multiple gene expressions in these tissues. For liver gene expression (Fig. 5A-D), there was a main effect of genotype (*p* < 0.05) and a main effect of diet (*p* < 0.05) for *Acadl* (Fig. 5A). Post hoc analysis showed that *Alb*^−/−^ had higher *Acadl* gene expression than *Alb*^+/−^ and WT (*p* < 0.05), and *Acadl* gene expression was also significantly higher in *Alb*^+/−^ than in WT (*p* < 0.05). Mice on the HFD had higher *Acadl* gene expression than mice on LFD. There was no genotype-by-diet interaction for *Acadl* gene expression. There was neither genotype-by-diet nor a main effect of genotype for *Acs*, *Glut2*, and *Tlr4*. However, there was a main effect of diet for *Acs* and *Glut2* (*p* < 0.05) (Fig. 5B, C). We also measured gene expression related to energy substrate metabolism in adipose tissue (Fig. 6). There was neither a significant main effect of genotype nor genotype-by-diet interaction for *Acadl* and *Acox1* gene expression in adipose tissue. However, there was a trend toward group differences for *Acadl*, with the p-value for the main effect of genotype being 0.06. There was a main effect of diet (*p* < 0.05) for *Acadl* and *Acox1* gene expression in adipose tissue, with mice on the HFD exhibiting higher *Acadl* and *Acox1* gene expression than mice on the LFD (Fig. 6B, C). There was a main effect of genotype (*p* = 0.05) and a main effect of diet (*p* < 0.05) for adiponectin (Fig. 6A). Post hoc analysis showed that *Alb*^−/−^ mice exhibited significantly higher adiponectin gene expression compared to WT mice (Fig. 6A). There was no genotype-by-diet interaction. Consistent with higher adiponectin gene expression in adipocytes, the ELISA measurement of plasma adiponectin concentration revealed that there was a main effect of genotype (*p* < 0.05). Post hoc analysis showed that *Alb*^−/−^ mice had higher plasma adiponectin concentration than *Alb*^+/−^ and WT mice(*p* < 0.05), and plasma adiponectin levels were also significantly higher in *Alb*^+/−^ than in WT (*p* < 0.05) (Fig. 7).

**Fig. 8:**
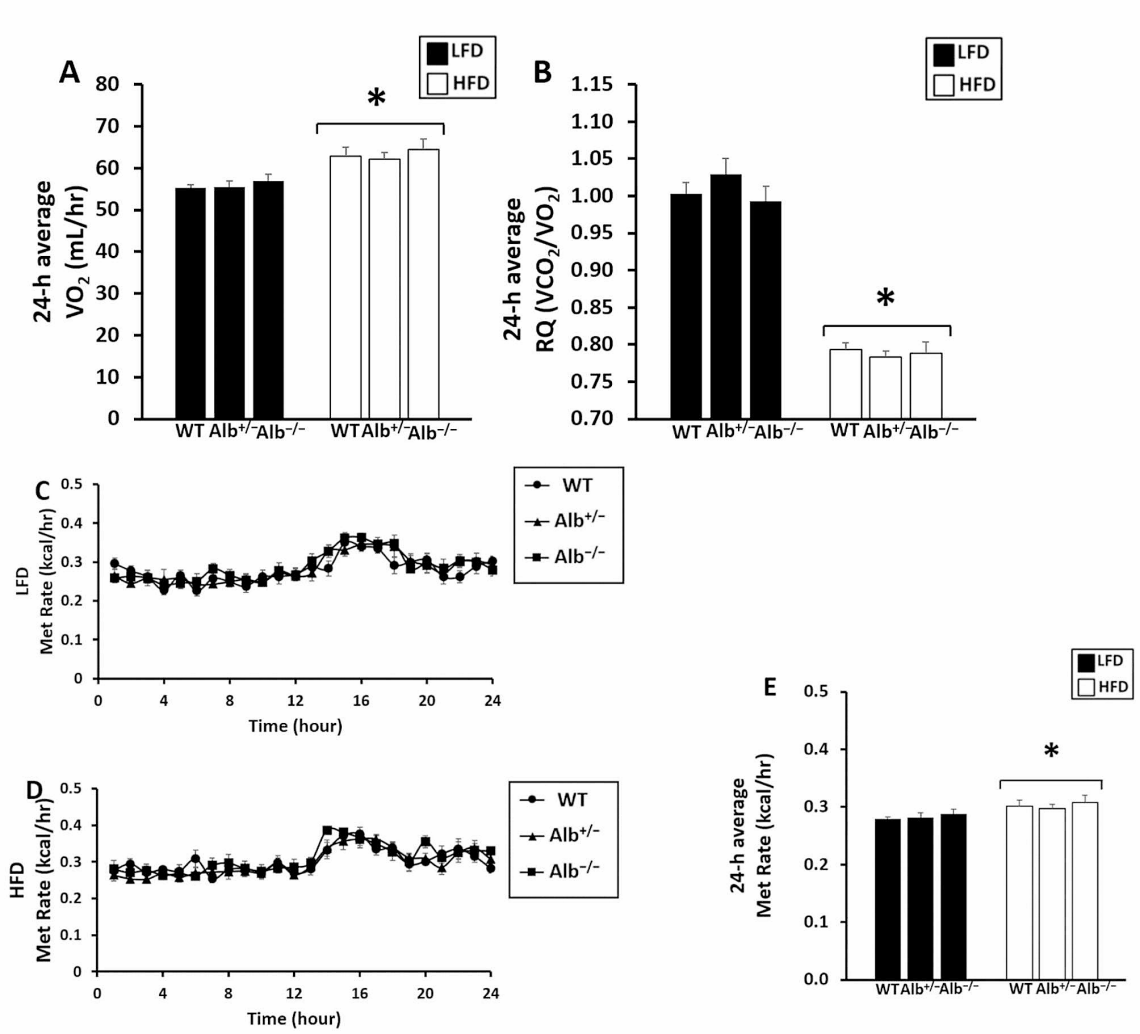
Fuel oxidation results. Oxygen consumption (VO_2_) expressed as the average over 24 h (**A**) and respiratory quotient (RQ) expressed as the average over 24 h (**B**); time course of metabolic rate over 24 h (**C**-**D**) and metabolic rate expressed as the average over 24 h (**E**). * HFD different from LFD (*p* < 0.05). *n* = 5 − 7. Analysis of RQ by ANOVA. Analysis of VO_2_ and metabolic rate by ANCOVA with covariate adjustment with body weight. The values are expressed as mean and standard error

Since FFAs are a metabolic fuel and are suppressed in *Alb*^−/−^ mice, indirect calorimetry was used for the evaluation of RQ and metabolic rate. In the indirect calorimetry analysis (Fig. 8A-E), a main effect of diet (*p* < 0.05) was observed for RQ, VO_2_, and metabolic rate. RQ was lower in mice on HFD than mice on LFD. The metabolic rate was higher in mice on HFD. The RQ was slightly above 1 in mice on LFD, likely indicating de novo lipogenesis. There were neither main effects of genotype nor diet-by-genotype interaction for these indirect calorimetry results.

**Fig. 9:**
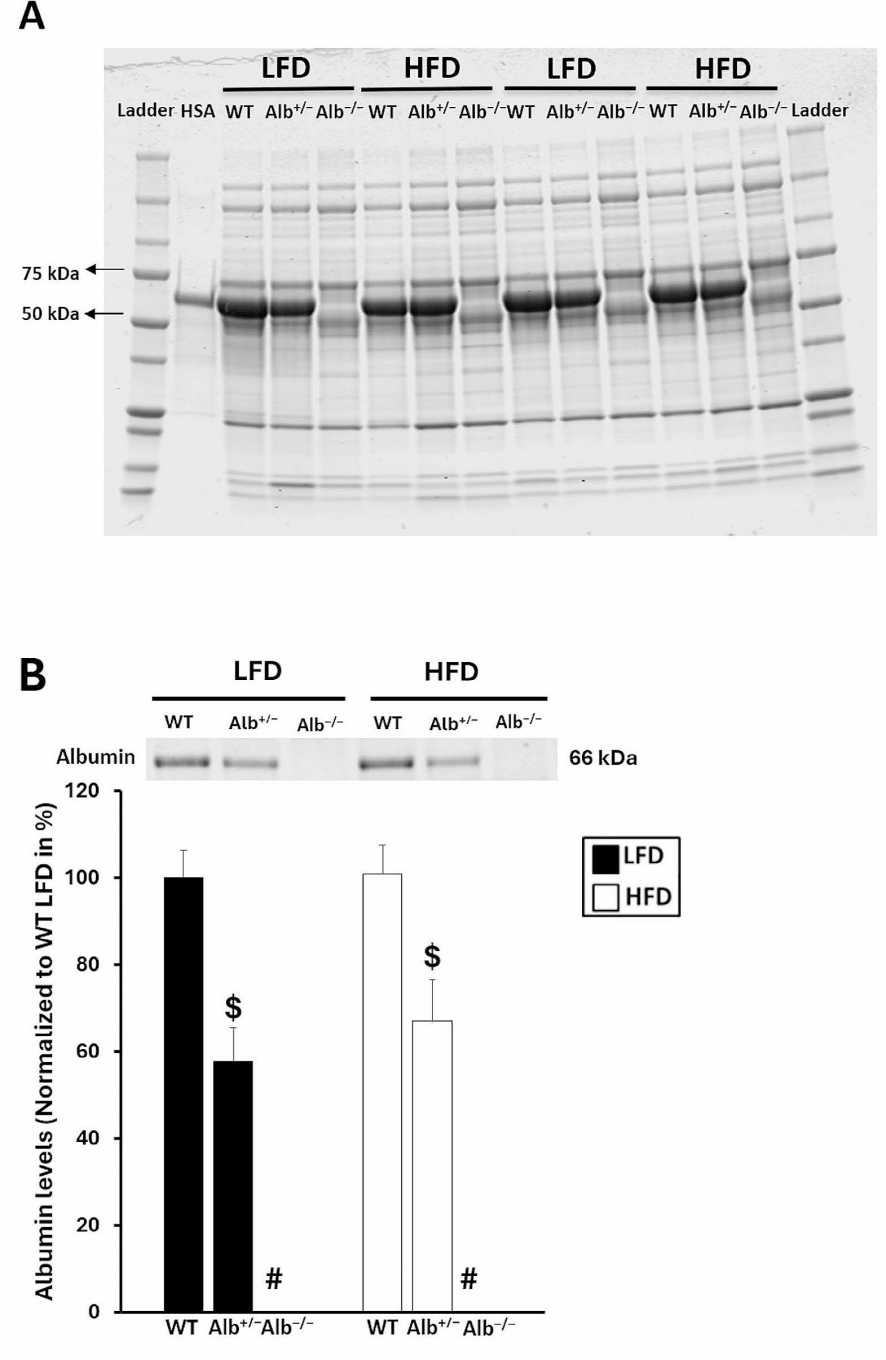
Gel electrophoresis analysis of plasma. The absence of albumin alongside presence of proteins at other molecular weights in Alb^−/−^ mice was confirmed by SDS-PAGE with Coomassie staining, through analysis of approximately 0.5 µL plasma per lane (**A**). Human Serum Albumin (HSA) was loaded as a molecular weight reference, corresponding to albumin’s molecular weight of approximately 66 kD. The relative differences between groups for albumin expression was assessed by SDS-PAGE with Coomassie staining, through analysis of approximately 0.02 µL plasma per lane, with densitometry quantitation of the band at the albumin molecular weight (**B**). ^#^ Alb^−/−^ different from Alb^+/−^ and WT within a diet (*p* < 0.05), ^$^ Alb^+/−^ different from WT and Alb^−/−^ within a diet (*p* < 0.05). WT group on the LFD is set to a value of 100%. *n* = 2 for panel **A** and *n* = 4 for panel **B**. Analysis of albumin expression was by ANOVA

Plasma protein molecular weight distribution was qualitatively visualized by SDS-PAGE (Fig. 9A), and in a separate analysis the plasma albumin abundances were estimated to determine how albumin expression may have varied between *Alb*^−/−^, *Alb*^+/−^, and WT mice (Fig. 9B). Through the approximation of albumin expression based upon quantitating the protein bands at the albumin molecular weight (~ 60–70 kD), we found a significant main effect of genotype with no significant main effect of diet or interaction. The post hoc analysis indicated that all three genotypes were statistically different from each other. Based on Coomassie staining technique, albumin levels were approximately 38% lower in *Alb*^+/−^ than in WT mice (42% lower in LFD and 33% lower in HFD) (Fig. 9B).

## Discussion

The results presented in this report extend our understanding of metabolic regulation in numerous ways. We discovered that the modest reduction in albumin expression in *Alb*^+/−^ mice is not sufficient to noticeably reduce plasma FFA concentration, while the full repression of albumin expression in *Alb*^−/−^ mice was sufficient to markedly reduce FFA, even when the mice were subjected to a HFD. With some minor exceptions, overall the phenotype of *Alb*^+/−^ mice was very similar to that of WT mice, while *Alb*^−/−^ mice exhibited a strikingly different phenotype from these other groups. As it is expected that female mice respond differently to obesogenic diets in comparison to males, here we studied females and discovered that even on a HFD the impacts of complete albumin deficiency led to improved glucoregulation. We discovered elevated adiponectin expression in albumin knockout mice, as well as changes in liver gene expression that indicate hepatic sensing of the metabolic state of albumin deficiency. The implications of these findings are discussed below.

One of the most fundamentally important questions to address in the present investigation was to what extent different levels of serum albumin can alter the concentration of plasma FFA. First, it was important to characterize the serum albumin concentration in WT, *Alb*^+/−^, and *Alb*^−/−^ mice. By gel electrophoresis, it was clear that the knockout of albumin in Alb^−/−^ mice is essentially complete, as we have reported previously [11, 12]. Others had reported that serum albumin would be reduced by approximately 30% in *Alb*^+/−^ compared to WT [14], but it was important for us to determine this value for the mice in our study. In general agreement, we found that the apparent albumin levels in plasma were approximately 30–40% lower in *Alb*^+/−^ than in WT mice. Next, it was of interest to determine if this modest reduction in serum albumin in *Alb*^+/−^ mice is sufficient to reduce the plasma FFA concentration. We discovered here that plasma FFA concentration was similar between *Alb*^+/−^ and WT mice, but substantially reduced in *Alb*^−/−^. Thus, a marked reduction in serum albumin expression is required to reduce plasma FFA. This can be understood from the fact that albumin expression is typically around a concentration of 0.2–0.4 mM in mice, and each albumin molecule can bind seven FFA. Therefore, the carrying capacity of serum albumin may be up to even over 2 mM, far beyond typical plasma FFA concentrations. Therefore, animals typically use less than half of their albumin-FFA binding capacity at any moment, explaining why a reduction in serum albumin beyond around 30 or 40% would be needed to impact the ability to transport FFA. It is known that plasma FFA concentration impacts insulin sensitivity. Infusion of heparin and a lipid emulsion in humans, a common technique for elevating plasma FFA concentration, rapidly reduces insulin sensitivity [26–31]. Therefore, it seemed likely that the unsuppressed plasma FFA concentration in *Alb*^+/−^ mice could limit any improvement in glucose tolerance.

*Alb*^−/−^ mice on HFD had lower plasma insulin, which is a common feature in groups with high insulin sensitivity. *Alb*^−/−^ mice also exhibited superior glucose tolerance and higher plasma adiponectin than the other genotypes, indicating the potential role of adiponectin in enhancing insulin sensitivity. *Alb*^−/−^ mice also exhibited lower NF-κB phosphorylation in the liver than *Alb*^+/−^ and WT suggesting lower levels of inflammation in hepatocytes which might also play a role in improved glucose tolerance in *Alb*^−/−^ mice. These hypotheses about a role of adiponectin or inflammation might ultimately be confirmed in future work. Importantly, *Alb*^+/−^ was metabolically similar to WT for these factors (plasma insulin, glucose tolerance, etc.), in agreement with *Alb*^+/−^ exhibiting a similar plasma FFA concentration to WT. The findings for glucose tolerance in *Alb*^−/−^ female mice on an obesogenic HFD are in agreement with our previous findings in male mice [12]. It could also be acknowledged, although it was not statistically significant, that there was a general trend for the body weight in *Alb*^−/−^ mice on HFD to be slightly lower than WT on the HFD, and thus one might wonder if the protection from glucose intolerance in this group was simply a result of lower body weight. We believe this was not the case, as *Alb*^−/−^ mice on a LFD also had better glucose tolerance than WT, even though the body weights did not show the trend seen on HFD. Thus, we expect that the nonsignificant trend for body weight differences between genotypes on the HFD is not a sufficient explanation for group differences in metabolic health. It was critical to study females, even after results on male mice had been published because it is known that the level of metabolic dysfunction in response to HFD differs between the sexes. Furthermore, it is important for data on both sexes to be published, when addressing various research questions, so that eventually the findings can be translated to benefit both women and men. Typically, female mice exhibit less weight gain and less induction of glucose intolerance on a HFD [19, 20, 32] with apparently superior homeostatic control. This superior homeostatic control in females vs. males led to the discovery in the present study that the HFD for 8 weeks did not induce much hepatic steatosis in females, and thus it was difficult to test in this study if albumin deficiency would reduce liver fat accumulation. However, it is also noteworthy that we previously showed that younger *Alb*^−/−^ female mice on a chow diet had significantly lower hepatic lipid accumulation compared to the WT mice at that same young age and on that same diet [11]. The reason that the present results did not recapitulate this finding could be explained by the differences in age or in diet. The diets in the present study (LFD and HFD) were purified diets which are distinct from chow diets. Nonetheless, despite limited ectopic lipid deposition in female mice in the current study, impressive group differences in glucose tolerance were observed. The potential mechanisms for improved glucose tolerance in *Alb*^−/−^ females are discussed below.

Although albumin deficiency could have some adverse effects, protective changes in lipid metabolism might counterbalance them; for instance, studies show lower plasma FFA levels in albumin knockout mice, which could be beneficial considering the health risks associated with higher FFA concentrations [11]. However, complete albumin deficiency, or specifically the genetic disease congenital analbuminemia, might elevate low-density lipoprotein cholesterol beyond normal ranges. Assessing this effect in patients is challenging due to the lack of direct comparisons to appropriately matched control subjects in the medical case reports literature. Despite widespread access to lipid-lowering medications, a significant portion of American adults suffer from dyslipidemia, suggesting that elevated serum lipid levels are prevalent globally, even in individuals expressing normal albumin levels. Nevertheless, it seems like albumin deficiency, or the associated decline in total plasma protein concentration, could exacerbate LDL cholesterol levels. Therefore, while some lipids like FFA may decrease in albumin deficiency, others such as LDL-C may increase [33].

To reiterate, complete albumin deficiency (*Alb*^−/−^) leads to lower blood glucose concentration in mice on both LFD and HFD, indicating the role of albumin deficiency in improving glucose metabolism even in obese mice. Albumin deficiency greater than observed here in the heterozygous albumin knockout mice (i.e., greater than ~ 40% suppression) is needed to observe the favorable effect of albumin deficiency on improved OGTT, as blood glucose levels were not different between *Alb*^+/−^ and WT mice. One of the factors that could control insulin sensitivity is adiponectin, and we found that plasma adiponectin concentration was higher in *Alb*^−/−^. It has been shown that adiponectin may increase insulin sensitivity by increasing the level of hepatic IRS-2 expression [34] or through enhanced activation of AMPK [35, 36]. In addition, adiponectin might improve insulin sensitivity through oxidative stress reduction and autophagy stimulation [37]. While there were no differences between genotypes for AMPK phosphorylation in the liver, albumin-deficient mice (*Alb*^−/−^) exhibited decreased levels of NF-κB activation. This could have been a result of the lower plasma FFA concentration, as FFA can lead to inflammatory signaling through toll-like receptor-4 (*Tlr4*). NF-κB is downstream of the *Tlr4* pathway [38]. Importantly, the expression of *Tlr4* was not different between genotypes, indicating that FFA concentration may drive *Tlr4* signaling rather than the expression level of that receptor controlling flux through the signaling pathway. It is known that blocking NF-κB in hepatocytes has a protective effect on the development of insulin resistance in mice [39], and it is possible that this response partially explains the present findings, in addition to the potential effects of adiponectin on glucoregulation. Furthermore, while it was not tested in the present study, liver glycogen concentration can also impact glucoregulation, and we previously discovered low glycogen in the liver of *Alb*^−/−^ mice [40]. Finally, we note that additional metabolic benefits in the liver may come from altered expression of the β-oxidation enzyme *Acadl*. It was interesting that even *Alb*^+/−^ mice exhibited a modest elevation of *Acadl* gene expression, while the response in *Alb*^−/−^ mice was more robust. Overall, changes in metabolic regulation clearly occur in albumin-deficient mice, although far more striking responses occur when albumin deficiency is more severe, as seen in the phenotype of *Alb*^−/−^ mice. Although studies on lipolysis inhibition and decreased FFA uptake by targeting fatty acid transporters have shown beneficial effects on improved glucose tolerance and decreased hepatic lipid accumulation, respectively, there is still no FDA-approved drug targeting FFA trafficking for patients with glucose intolerance and fatty liver disease. The present study demonstrates that in addition to studying lipolysis and membrane fatty acid transporters, FFA transport through the bloodstream is also critically important to be studied. Understanding this response can further our understanding of mammalian physiology. Studying proteins such as albumin that carry FFA in blood can also help the scientific community conceive of new potential strategies for reducing plasma FFA. For example, it may be that inhibiting the binding of FFA to albumin’s FFA binding sites in the future could interrupt FFA transport in the bloodstream, potentially leading to improved glucoregulation. Future work on therapeutics development and then clinical research could further explore this idea.

### Strengths and limitations

There are numerous strengths of the present investigation as well as some limitations. It is a strength that female mice were studied, as in the past, studies of male mice have predominated in the literature. It is also a strength that heterozygous gene knockout mice were compared to homozygous knockout mice, in order to understand if there was an intermediate phenotype when only one copy of the albumin gene is mutated; this led to a deeper understanding of the dose-response relationship between albumin and metabolism. It is also a strength that physiological data were combined with biochemical and molecular data, in order to carry out an integrative physiology investigation. It is a limitation that our sample size was limited to 5–7 mice per group; by increasing the sample size the effects of confounding factors such as epigenetic variability and environmental factors can be minimized. In addition, females were not studied simultaneously with males, as the comparison of the present data to our manuscript on males [12] is limited by comparing data from studies that were carried out at different points in time, and we have not determined the phenotype of male *Alb*^+/−^ mice. It is also a limitation that a single age was studied, as it is possible that the phenotype of albumin deficiency could be different at different ages; however, at the ages we have studied thus far, ~ 8 weeks [11] and ~ 18 weeks [12], both males and female *Alb*^−/−^ mice do exhibit superior glucose tolerance in comparison to WT mice, indicating that generally, the relationship between albumin deficiency and glucoregulation has been robust. In addition, more genes and protein expressions involved in lipid and glucose metabolism could be studied in the future.

There are multiple future directions for studying albumin deficiency and its beneficial effects on metabolic health. First, investigating the impact of albumin deficiency on other organs such as skeletal muscle could delve into the role of skeletal muscle in glucose uptake and improved glucoregulation. Second, how adipose tissue adapts in response to albumin deficiency could provide insights into metabolic regulation and potentially provide strategies for managing obesity-related disorders. Third, studying double knockout mouse models (e.g., albumin knockout and adiponectin knockout, or albumin knockout and NF-κB knockout) could potentially indicate whether the improved glucoregulation in albumin knockout mice is due to increased plasma adiponectin concentration or decreased hepatic phosphorylated NF-κB level. Fourth, future work on ovarian hormones may be warranted; studying ovariectomized mice (or mice with specific estrogen receptors knocked out) may shed light on the potential beneficial effect of albumin deficiency on metabolic health when the protective roles of estrogen are minimized. Continued research in such areas could help to elucidate the potential relationships between albumin expression and metabolic health, as well as the intricate mechanisms underlying albumin deficiency’s effects on the body.

## Conclusions

In summary, our results indicate that albumin deficiency in female mice reduces plasma FFA concentration and decreases blood glucose and insulin concentrations when mice are challenged with an obesogenic diet. The improved glucoregulation might in part be through increased plasma adiponectin concentration. The present results also provide a deeper understanding of heterozygous albumin knockout mice than had previously been appreciated. With this new information, it has become clear that a modest reduction in albumin expression is not sufficient to suppress the plasma FFA level or to alter glucose tolerance. To this point, the understanding of metabolism in female mice has been studied to a lesser extent than in males, yet sex is an important biological variable. With these new data, the relationship between serum albumin expression and the regulation of metabolism in female mice has been elucidated. The results extend our understanding of metabolism in relation to metabolic health.

## Data Availability

The datasets used and/or analyzed during the current study are available from the corresponding author upon reasonable request.
